# The Impact of Vaccinations Against Respiratory Infections on the Prognosis in Heart Failure Patients

**DOI:** 10.3390/vaccines12121321

**Published:** 2024-11-26

**Authors:** Berenika Jankowiak, Marta Wleklik, Marta Rosiek-Biegus

**Affiliations:** 1 Institute of Heart Diseases, Wroclaw Medical University, 50-556 Wroclaw, Poland; 2 Division of Research Methodology, Department of Nursing, Faculty of Nursing and Midwifery, Wroclaw Medical University, 50-556 Wroclaw, Poland; marta.wleklik@umw.edu.pl; 3 Institute of Internal Diseases, Wroclaw Medical University, 50-556 Wroclaw, Poland; marta.rosiek-biegus@umw.edu.pl

**Keywords:** heart failure, influenza vaccination, pneumococcal vaccination, COVID-19 vaccination, RSV vaccination, cardiovascular diseases

## Abstract

Heart failure (HF) affects 64 million people worldwide and is one of the most prevalent causes of hospitalization in adults. Infection is believed to be one of the potential triggers that may facilitate HF decompensation and the need for hospitalization. Therefore, it seems crucial to safeguard against such a situation. Vaccinations seem to be a very reasonable option. However, this remains an underutilized solution among HF patients. This review investigates the impact of available vaccinations, including influenza, COVID-19, pneumococcal, and RSV, on prognosis in specific HF populations only, as there are pathophysiological reasons to believe that this population of patients may benefit the most from the intervention. It will provide information about the safety profile of these vaccines and summarize the available evidence on their impact on hard clinical outcomes. In summary, this article will discuss the impact of preventive vaccinations against seasonal infections in the HF population.

## 1. Introduction

Heart failure (HF) is a multifaceted syndrome affecting 64 million people worldwide [1] and is the leading cause of hospitalization for individuals over 65 years of age [2,3,4]. The pathophysiology of the disease is complex and involves the background neurohormonal overactivity, compromised immune function, and chronic inflammatory state [5,6,7,8]. This not only affects the cardiovascular system but influences the function of other organs and systems, thereby further impacting daily functioning and significantly lowering the health-related quality of life (HRQoL) [9] of patients. This leads to numerous hospitalizations and a shortening of life [8,10,11,12,13].

In the general population, infections can cause thrombosis or exacerbation of atherosclerosis, which can result in myocardial infarction. The perfect example is COVID-19, which can cause dysfunction of endothelium and activation of platelets, which in turn leads to hypercoagulability and excessive inflammation state, which has prothrombotic potential. This pathophysiology can also trigger arrhythmia [14]. Influenza and COVID-19 increase the risk of acute myocardial infarction [15,16,17,18]. Infection is one of the potential trigger mechanisms that may disturb the fragile homeostasis of HF pathophysiology, which may lead to episodes of overt HF decompensation. On the other hand, the direct viral infection affecting the cardiomyocytes may lead to myocarditis and life-threatening clinical scenarios [19,20,21].

Moreover, most HF patients are frail; the disease itself, as well as co-morbidities and advanced age, make the population even more frail and prone to any infection-related complications [22,23,24]. Based on those assumptions, it is hypothesized that it’s important for patients affected by the disease to be vaccinated—especially prior to the infection season [25,26]. All of these factors pose a potential cause of HF development and progression in some scenarios. Infections can also pose significant dangers to a patient’s already exhausted body, potentially leading to severe conditions, such as sepsis or septic shock [27].

A study conducted by Alon and Stein et al. showed that patients with HF hospitalized due to respiratory tract infections or sepsis/bacteremia have a much higher 30-day mortality rate than patients hospitalized for other infections [28]. The same study also showed that upper respiratory tract infections are one of the most common reasons for hospitalization in HF patients, accounting for as much as 52.6% of hospitalization causes in this population [28]. It is interesting, therefore, that the vaccination rate among the HF population is relatively low [29].

All those facts set the stage for this article, in which we present a review of the available data summarizing the impact of respiratory infection prevention by influenza, COVID-19, respiratory syncytial virus (RSV), and Streptococcus pneumoniae vaccinations on the outcomes of the HF populations. We will also present the safety profile of these vaccinations.

## 2. Results

### 2.1. Influenza

#### 2.1.1. The Risk Related to Influenza Infection

The influenza virus, belonging to Orthomyxoviridae, causes acute respiratory tract infection with secondary complications, including myocarditis, pericarditis, acute kidney injury, secondary bacterial pneumonia, exacerbation of chronic diseases, such as COPD or HF, as well as Reye syndrome or Guillain–Barré syndrome. Influenza affects people in all age groups and is associated with high mortality, especially among higher-risk groups like elderly patients or those with severe comorbidities. Due to point mutations (antigenic drift), there are annual epidemics, creating the need for seasonal updates in the vaccines against this virus [30,31,32].

Clinical research by Panhwar et al. revealed that influenza infection is linked to a higher risk of in-hospital morbidity and in-hospital mortality among HF patients [33].

#### 2.1.2. The Reported Benefit of Influenza Vaccination in HF Population

In a study by Loeb et al., conducted in Asia, the Middle East, and Africa, analyzing the impact of influenza vaccination in reducing adverse vascular events in 5129 HF patients, the comparison was made between the effect of this vaccine and all-cause hospitalizations, pneumonia, all-cause death, cardiovascular death, non-fatal myocardial infarction, non-fatal stroke, and HF hospitalization. This study was divided into the infectious and non-infectious seasons. During the non-infectious season, differences between the study group receiving vaccination and the controls receiving placebo were not significant, with a lower number of pneumonia cases in vaccinated individuals compared to controls. During the infectious season, a reduction in the number of all-cause deaths, cardiovascular deaths, and pneumonia was observed in vaccinated patients. The vaccinated cohort had the highest reduction in pneumonia [34].

A meta-analysis by Poudel et al. [35] conducted on studies involving 16,751 patients showed a significant reduction in mortality risk in HF patients vaccinated against influenza within a year of vaccination (HR, 0.69; 95% CI, 0.51–0.87), as pictured in Table 1, index 2. The same meta-analysis involving 9055 patients showed a lower risk of hospitalization among vaccinated patients compared to the unvaccinated group (HR, 0.62; 95% CI, 0.00–1.23), which was, however, statistically not significant.

The study by Modin et al. conducted on 134,048 individuals also showed a decrease in mortality among HF patients vaccinated annually compared to unvaccinated individuals by 19%, both in the case of all-cause death risk (HR, 0.82; 95% CI, 0.81–0.84, *p* < 0.001), and in the case of cardiovascular death (HR, 0.82; 95% CI, 0.81–0.84, *p* < 0.001). Vaccination in the HF population was also associated with a lower risk of hospitalization due to influenza or pneumonia (HR, 0.96; 95% CI, 0.93–0.98, *p* = 0.002) [36].

Kopel et al., after analyzing vaccinated compared to not-vaccinated HF patients assigned to the HFSIS (the heart failure survey in Israel) registry, showed that in-hospital and 1-year mortality rates were HR, 0.71; 95% CI 0.42–1.18, *p* = 0.19 and HR, 0.81; 95% CI 0.66–0.99, *p* = 0.04, respectively. Particular significance was found in the observed risk of 4-year mortality, which amounted to HR, 0.83; 95% CI, 0.73–0.95, *p* = 0.006 [37].

The PARADIGM-HF-Trial with 8099 participants showed us that influenza vaccination is related with reduced risk for all-cause death (HR, 0.81; 95% CI 0.67–0.97, *p* = 0.015). It also gave us information about the percentage of vaccinated residents for each country—the data is presented in Table 2 [38].

The meta-analysis of Gupta et al. was focused on reducing adverse events by influenza vaccine. It demonstrated that the risk of all-cause cardiovascular-related mortality was significantly reduced, but all-cause hospitalization was higher among vaccinated patients with heart failure [41].

In the clinical trial presented by Vardeny et al. [44], influenza vaccines—high dose trivalent and standard dose quadrivalent—were considered separately among patients with high-risk cardiovascular diseases. The comparison showed that the high dose trivalent vaccine did not significantly reduce the risk of all-cause death and cardiopulmonary hospitalizations.

In Table 2, we presented a comparison of influenza vaccination coverage across specific countries [38,39]. We compared the two extremes presented in the table—Poland and Spain—based on the statistical data we gathered. In Poland, in 2021, the prevalence was 2.63%, with as many as 205,000 hospitalizations due to heart failure (HF); while in Spain, the prevalence in 2019 was close to 2%, with an incidence rate of 2.78 per 1000. This difference was influenced not only by vaccination coverage but by various other factors, such as differences in the age structure of the populations studied and genetic differences. However, considering the aforementioned research, we can infer that vaccination is significant in protecting HF patients [43,45].

#### 2.1.3. The Safety Data of Influenza Vaccination

The safety profile of the influenza vaccine also needs to be pointed out. There is no increased risk of side effects, such as Guillain–Barré syndrome, seizures, encephalitis, or anaphylaxis following vaccination in HF population [46]. The vaccination is recommended directly in current ESC guidelines, class IIa, level of evidence B [9]. Importantly, although vaccination is recommended to reduce the risk of HF hospitalizations, it is also mentioned as an effective intervention to prevent the development of HF. No specific recommendation is made with respect to the type of vaccine [47].

### 2.2. COVID-19

#### 2.2.1. The Risk Related to COVID-19 Infection in Heart Failure Population

Severe acute respiratory syndrome coronavirus 2 (SARS-CoV-2) is a pathogen from the Coronaviridae family, first registered in China in 2019. This contagious virus has caused millions of confirmed widespread cases and deaths [48,49]. Patients with conditions such as heart failure are at an especially elevated risk of morbidity and mortality caused by this pathogen [50,51,52,53]. COVID-19 itself may also result in cardiac disorder, presenting diverse ventricular dysfunctions (including myocarditis) [51].

The vast majority of patients experience this infection mildly, but some prone patients with severe illnesses, such as those with HF [50], may develop what is known as a cytokine storm, an excessive immune system response that can result in severe respiratory failure [54].

There have also been reported cases of pericarditis, myocardial infarction, Takotsubo cardiomyopathy, and arrhythmias developing as a result of COVID-19 infection, as well as cardiogenic shock related to the infection, which may lead to myocardial dysfunction themselves [19,55,56,57,58,59,60,61,62]. Paradoxically, lifestyle changes related to the pandemic and lockdowns decreased emergency admissions, and a lower risk of HF hospitalizations, compared to the pre-pandemic timeframe [63,64,65,66,67,68,69]. Moreover, the self-isolation of many patients and in-depth knowledge/fear regarding the risks of infection may also influence the lower rates of hospitalizations.

On the other hand, we may argue that this phenomenon is more complex than that. The other possible explanation for the lower emergency admission rates may be related to the assumption that patients (especially those at the highest risk) ignored severe conditions (like clinical signs of decongestion or chest pain) to prevent hospitalization, as the highest risk of acquiring the COVID infection was in the hospital. This was associated with higher out-of-hospital mortality, worse performance indicators, and higher in-hospital mortality (as patients admitted were, in general, in more advanced stages of the disease) [70].

#### 2.2.2. The Reported Benefit of COVID-19 Vaccination in HF Population

The study by Sinder-Pedersen et al., based on Danish registries included patients (each cohort consisting of 50,893 patients), compared the risk of 90-day all-cause death, 90-day in-hospital admission for HF, 90-day risk of venous thromboembolism, 90-day risk of myocarditis, and 90-day risk of pneumonia in HF patients vaccinated against COVID-19 (data from 2021) with the risk in HF patients unvaccinated against COVID-19 (data from 2019). The risk difference was −0.33% [95% CI, −0.52% to −0.15%] for all-cause death, favoring vaccinated individuals; the difference in the risk of HF decompensation within 90 days was not significant and amounted to 0.02% [95% CI, −0.11% to 0.15%]; however, if the patient was hospitalized within 90 days, the risk difference of HF decompensation was −1.91% [95% CI, −5.25% to 1.43%] with an advantage for vaccinated individuals. The risk difference for pneumonia was significantly lower in vaccinated individuals −0.64% [95% CI, −0.81% to −0.49%]. The risk difference for venous thromboembolism was minimal and amounted to non-significant −0.02% [95% CI, −0.05% to 0.01%] in favor of vaccinated individuals (Table 1) [42].

Studies indirectly support the benefit of COVID-19 vaccination in relation to HF. This vaccination reduces the risk of cardiovascular outcomes due to COVID-19 infection, such as myocardial infarction [16,17], which can trigger HF development and decompensation.

#### 2.2.3. The Safety Data of COVID-19 Vaccination

It has been shown that mRNA vaccines are associated with an increased risk of myocarditis and pericarditis, especially in males aged 12–17 years. The pathophysiology of myocarditis and pericarditis related to COVID-19 (as well as other viruses) is poorly understood. It is hypothesized that some COVID-19-related myocarditis involves direct viral damage to cardiomyocytes through SARS-CoV-2 binding to ACE2 receptors, triggering apoptosis and necrosis. The infection induces a strong inflammatory response, including a cytokine storm (e.g., IL-6, IL-1β, TNF-α), endothelial dysfunction, and microvascular thrombosis, which exacerbate myocardial injury. Autoimmune mechanisms and subsequent fibrosis lead to long-term cardiac remodeling, reducing contractility, and increasing the risk of heart failure and arrhythmias.

Long COVID, on the other hand, refers to a range of persistent symptoms and complications that occur months after the acute infection. Its pathophysiology is multifactorial, involving chronic inflammation, immune dysregulation, and endothelial dysfunction, leading to tissue damage and impaired recovery. Viral persistence in certain tissues may also contribute to ongoing symptoms. Additionally, mitochondrial dysfunction and altered autonomic regulation (dysautonomia) are thought to play a role in fatigue, cardiovascular instability, and neurological manifestations. Notwithstanding, the vast majority of patients experience mild infection and do not need admission to the hospital; negligence of these conditions is dangerous. They can occur as patients, usually young men, with pain in the chest, heart palpitations, or shortness of breath during the first 7 days after vaccination [52]. These vaccines may also lead to such adverse events as lymphadenopathy or facial nerve palsy [15,56,57,58,59,61,62,63,64,65,66,67,68,69,70,71,72,73,74]. Autopsy studies have shown a causal relationship between vaccination and the abovementioned complications [66,75,76].

However, the study by Barda et al. [77] showed that, while the post-vaccination risk is significantly increased (RR, 3.24; 95% CI, 1.55–12.44), it is much lower than the risk after COVID-19 infection (RR, 18.28; 95% CI, 3.95–25.12). It is notable that a complete vaccination course provides protection not only for HF patients, but for patients undergoing treatment for myocardial infarction [78]. Be that as it may, being simultaneously vaccinated and having post-infection immunization appear to predispose patients with myocardial infarction with ST elevation to evolving cardiovascular conditions, such as cardiogenic shock or severe heart failure [20]. The protection and effectiveness of the vaccination can be elevated using boosters [53].

The most commonly used vaccines were two-dose Pfizer–BioNTech (BNT162b2), Oxford–AstraZeneca (AZD1222), Moderna (mRNA-1273), and single-dose Johnson & Johnson (Ad26.COV2.S), with subsequent booster doses being desirable [79]. Furthermore, a meta-analysis from 2023 indicates that the number of serious adverse events in older individuals after this vaccination is very low despite the occurrence of post-vaccination symptoms, such as pain or fever [80].

Cases, such as immune thrombotic thrombocytopenia (TTS), cerebral venous sinus thrombosis, VTE, TIA and stroke, acute myocardial infarction, arrhythmia, and large-vessel vasculitis, have been reported following COVID-19 vaccination, but the risk is disproportionately low compared to the risk following the infection itself [55,81,82,83]. Similarly, notwithstanding confirmed myocarditis and pericarditis following COVID-19 vaccination cases, the protective role occurred to overcome the risks, especially among the elderly [84].

### 2.3. Pneumococcal Vaccination

#### 2.3.1. The Risk Related to Pneumococcal Infection

Streptococcus pneumoniae is a gram-positive bacteria that is a common cause of many pneumococcal infections, such as pneumonia, otitis media, meningitis, and sepsis. It is a serious threat, especially for children, the elderly, and immunocompromised patients [85]. The direct data on the HF population is scarce, but based on the HF pathophysiology and the fact that the HF population is more likely to develop infection-related complications, it is highly advisable for HF patients to prevent those pathogens [86,87].

#### 2.3.2. The Reported Benefit of Pneumococcal Vaccination

Pneumococcal vaccination, similar to influenza vaccination, is recommended in the recent ESC guidelines for managing heart failure (class IIa, level of evidence B) [9]. However, prospective randomized controlled trials are lacking to directly confirm the impact of this vaccination on patients with heart failure [88]. Available studies are not unanimous, so this cannot be conclusively stated. The meta-analyses by Marra et al. and Marques Antunes et al. demonstrated that pneumococcal vaccination was associated with a reduction in the risk of cardiovascular events, all-cause mortality, and myocardial infarction, in the former, and all-cause mortality in patients at very high cardiovascular risk, in the latter [89,90]. However, it is important to note that the populations examined in the meta-analyses were not limited to heart failure.

In the huge observational study by Bhatt et al. analyzing 313,761 patients from late 2012 to early 2017 (from the Get With The Guidelines–HF Registry), it was shown that pneumococcal and/or influenza vaccination does not affect the clinical outcomes of HF patients [40]. This may represent a true lack of the protective effect of the pneumococcal vaccination in the HF population or the high competing risk in the population that significantly reduces the observed benefit of the intervention.

#### 2.3.3. The Safety Data of Pneumococcal Vaccination

PCV13 and PPSV23 vaccines, most used against pneumococci, are safe for adults, including those over 65. The most reported side effects were headache, myalgia, arthralgia, pain at the injection site, and fatigue [91,92,93].

### 2.4. RSV Vaccination

#### 2.4.1. The Risk Related to RSV Infection

Respiratory syncytial virus (RSV), belonging to the Paramyxoviridae family, is commonly known for childhood infections. Like pneumococci, it also poses a threat to adults with reduced immunity and the elderly, especially those with cardiovascular diseases such as HF [94]. RSV infection is associated with an increased risk of acute heart failure, and patients with chronic HF are at a heightened risk for RSV infection [95,96].

In a cross-sectional study of more than 6200 adults (>50 years old) observed over five RSV seasons, nearly one-quarter experienced an acute cardiac event (most frequently AHF). The risk of severe outcomes was nearly twice as high in patients with acute cardiac events compared with patients who did not experience an acute cardiac event [95].

Hospitalization due to RSV infection may be associated with or complicated by outcomes such as acute respiratory illness, arrhythmias, myocardial infarction, acute heart failure, Intensity Care Unit admission, receipt of invasive mechanical ventilation, or in-hospital death [97]. In the study conducted by Loubet et al., 19% of patients also presented acute heart failure during hospitalization for RSV [98]. New diagnostic techniques may lead to significant improvements in the identification of the infection [96].

#### 2.4.2. The Benefit of RSV Vaccination

The Arexvy (GSK) and Abrysvo (Pfizer) vaccines have been available for individuals aged ≥60 years only since 2023 and are not currently included in ESC recommendations, as little is known about them in the context of HF.

#### 2.4.3. The Safety Data of RSV Vaccination

The study by Biegus et al. [99] examined the early safety profile of simultaneous vaccination with Arexvy and Vaxigrip Tetra vaccines. In this prospective study, 105 patients received both vaccines, and only 6% of patients reported symptoms that interfered with their daily functioning. There were also no deaths or hospitalizations due to HF decompensation or any other serious adverse events within a week after vaccination.

## 3. Conclusions

Vaccination against respiratory system infections in HF patients remains a topic that needs to be discovered further. Notwithstanding the fact that HF patients are at high risk of both infections and their complications, there are relatively few prospective, randomized studies that clearly address their impact on HF prognosis. Large-scale, high-quality data are extremely needed; as important as the effect of vaccinations is, it is probably difficult to demonstrate, due to large numbers of competing risks in HF cohorts. Thus, the “pure” protective effect of the intervention is blunted and may be lost, especially in small samples.

Influenza vaccination is one of the best-studied in the HF population, and therefore, it is recommended in many sources, with its benefits being evident. The early safety profile of this vaccine has also been investigated in combination with simultaneous vaccination against RSV and influenza and appears promising. It would be particularly desirable to conduct RCTs on the impact of pneumococcal and RSV vaccinations in individuals with HF, as the definitive evidence in the group is missing.

## Figures and Tables

**Table 1:** Impact of individual vaccines in HF populations on selected outcomes.

**Index**	**Study**	**Vaccine**	**Number of Patients**	**Outcome**	**Hazard Ratio (95% CI)**	***p*-Value**
1	Loeb et al. [34]	Influenza	2560 vaccinated	Death-all cause	0.90 (0.79–1.03)	0.13
				Death-cardiovascular	0.89 (0.77–1.04)	0.13
				Hospitalization for heart failure	0.88 (0.74–1.05)	0.15
				Pneumonia	0.58 (0.42–0.80)	0.0006
2	Poudel et al. [35]	Influenza	82,354	Death-all cause	0.69 (0.51–0.87)	Not included
				Hospitalization for heart failure	0.62 (0.00–1.23)	Not included
3	Modin et al. [36]	Influenza	134,048	Death-all cause	0.82 (0.81–0.84)	<0.001
				Death- cardiovascular	0.82 (0.81–0.84)	<0.001
				Hospitalization due to influenza or pneumonia	0.96 (0.93–0.98)	0.002
4	Kopel et al. [37]	Influenza	1964 (501 vaccinated)	In-hospital mortality	0.71 (0.42–1.18)	0.19
				1-year mortality	0.81 (0.66–0.99)	0.04
				4-year mortality	0.83 (0.73–0.95)	0.006
5	Vardeny et al. [38]	Influenza	8099 (1769 vaccinated)	All-cause death	0.81 (0.67–0.97)	0.015
6	Gotsman et al. [39]	Influenza	6435 (4440 vaccinated)	All-cause mortality	0.77 (0.65–0.91)	<0.01
				Death and cardiovascular hospitalizations	0.83 (0.76–0.90)	<0.001
7	Bhatt et al. [40]	Pneumococcal infection	313,761	Pneumococcal vaccination does not affect the clinical outcomes of HF patients		
**Index**	**Study**	**Vaccine**	**Number of Patients**	**Effect**	**Risk Ratio (95% CI)**	***p*-Value**
8	Gupta et al. [41]	Influenza	247,842	All-cause mortality	0.75 (0.71–0.79)	<0.0001
				Cardiovascular-related mortality	0.77 (0.73–0.81)	<0.0001
				All-cause hospitalization	1.24 (1.13–1.35)	<0.0001
**Index**	**Study**	**Vaccine**	**Number of Patients**	**Effect**	**Risk Difference (95% CI)**	
9	Sinder-Pedersen et al. [42]	COVID-19	101,786 (50,893 vaccinated)	90-day risk all-cause death	−0.33% (−0.81% to −0.49%) with an advantage for vaccinated	
				90-day risk in-hospital admission for HF	0.02% (−0.11% to 0.15%) with an advantage for not vaccinated	
				90-day risk of venous thromboembolism	−0.02% (−0.05% to 0.01%) with an advantage for vaccinated	
				90-day risk pneumonia	−0.64% (−0.81 to 0.49%) with an advantage for vaccinated	

**Table 2:** Influenza vaccination coverage in specific countries.

Trial/Source	Country	Year	Vaccination	65 Years or over Vaccinated HF Population (%)	Total Vaccinated HF Population (%)	Clinical Trial Vaccinated Population (%)
OECD Health Statistics 2024 [43]	Poland	2022	Influenza	8.6	-	-
	Germany	2022	Influenza	43.3	-	-
	France	2023	Influenza	56.2	-	-
	Czech Republic	2023	Influenza	25.4	-	-
	Norway	2023	Influenza	64.2	-	-
	Spain	2023	Influenza	68.5	-	-
	Poland	2023	Influenza	-	5.5	-
The PARADIGM-HF Trial—Vardeny et al. [38]	Netherlands	2016	Influenza	-	-	77.5
	Great Britain					77.2
	Belgium					67.5
	Asia					2.6
	North America					52.8
	Poland					4.6
	United States					55.1
	Sweden					55.2

## Abbreviations

HF—heart failure; RSV—respiratory syncytial virus; HRQoL—health-related quality of life; COPD—chronic obstructive pulmonary disease; HFrEF—heart failure with restricted ejection fraction; HfmrEF—heart failure with mild restricted ejection fraction; HFpEF—heart failure with preserved ejection fraction.
